# Anaerobic Antibiotic Coverage in Aspiration Pneumonia and the Associated Benefits and Harms: A Retrospective Cohort Study

**DOI:** 10.1016/j.chest.2024.02.025

**Published:** 2024-02-20

**Authors:** Anthony D. Bai, Siddhartha Srivastava, Geneviève C. Digby, Vincent Girard, Fahad Razak, Amol A. Verma

**Affiliations:** a Division of Infectious Diseases, Department of Medicine, Queen’s University, Kingston, ON, Canada; b Division of General Internal Medicine, Department of Medicine, Queen’s University, Kingston, ON, Canada; c Division of Respirology, Department of Medicine, Queen’s University, Kingston, ON, Canada; d Internal Medicine Residency Program, Department of Medicine, Queen’s University, Kingston, ON, Canada; e Department of Medicine, University of Toronto, Toronto, ON, Canada; f Institute of Health Policy, Management and Evaluation, University of Toronto, Toronto, ON, Canada; g Li Ka Shing Knowledge Institute, St. Michael’s Hospital, Unity Health Toronto, Toronto, ON, Canada

**Keywords:** antibiotic treatment, aspiration pneumonia, mortality

## Abstract

**Background:**

Antibiotics with extended anaerobic coverage are used commonly to treat aspiration pneumonia, which is not recommended by current guidelines.

**Research Question:**

In patients admitted to hospital for community-acquired aspiration pneumonia, does a difference exist between antibiotic therapy with limited anaerobic coverage (LAC) vs antibiotic therapy with extended anaerobic coverage (EAC) in terms of in-hospital mortality and risk of *Clostridioides difficile* colitis?

**Study Design and Methods:**

We conducted a multicenter retrospective cohort study across 18 hospitals in Ontario, Canada, from January 1, 2015, to January 1, 2022. Patients were included if the physician diagnosed aspiration pneumonia and prescribed guideline-concordant first-line community-acquired pneumonia parenteral antibiotic therapy to the patient within 48 h of admission. Patients then were categorized into the LAC group if they received ceftriaxone, cefotaxime, or levofloxacin. Patients were categorized into the EAC group if they received amoxicillin-clavulanate, moxifloxacin, or any of ceftriaxone, cefotaxime, or levofloxacin in combination with clindamycin or metronidazole. The primary outcome was all-cause in-hospital mortality. Secondary outcomes included incident *C difficile* colitis occurring after admission. Overlap weighting of propensity scores was used to balance baseline prognostic factors.

**Results:**

The LAC and EAC groups included 2,683 and 1,316 patients, respectively. In hospital, 814 patients (30.3%) and 422 patients (32.1%) in the LAC and EAC groups died, respectively. *C difficile* colitis occurred in five or fewer patients (≤ 0.2%) and 11 to 15 patients (0.8%-1.1%) in the LAC and EAC groups, respectively. After overlap weighting of propensity scores, the adjusted risk difference of EAC minus LAC was 1.6% (95% CI, –1.7% to 4.9%) for in-hospital mortality and 1.0% (95% CI, 0.3%-1.7%) for *C difficile* colitis.

**Interpretation:**

We found that extended anaerobic coverage likely is unnecessary in aspiration pneumonia because it was associated with no additional mortality benefit, only an increased risk of *C difficile* colitis.

## Graphical Abstract

**Figure:**
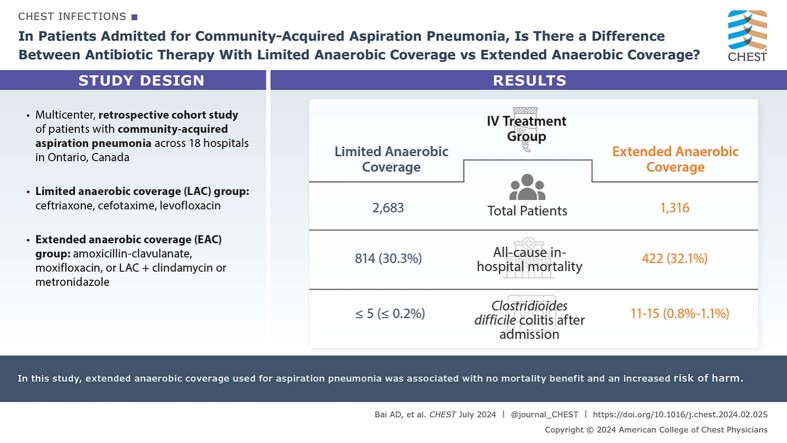

**Take-home Points**

**Study Question:** In patients admitted to hospital for community-acquired aspiration pneumonia, does a difference exist between antibiotic therapy with limited anaerobic coverage and extended anaerobic coverage in terms of in-hospital mortality and risk of *Clostridioides difficile* colitis?

**Results:** In this retrospective cohort study of 3,999 patients, the extended anaerobic coverage group showed similar in-hospital mortality and a higher risk of *C difficile* colitis when compared with the limited anaerobic coverage group.

**Interpretation:** We found that extended anaerobic coverage likely is unnecessary in aspiration pneumonia because it was associated with no mortality benefit and an increased risk of harm.

Aspiration pneumonia is a bacterial lung infection that results from a large-volume aspiration of oropharyngeal and gastric contents.^1^ It accounts for 5% to 15% of community-acquired pneumonia (CAP)^1^ cases and is associated with high mortality.^2^^,^^3^ In a cohort study of 4,263 hospitals, the 30-day mortality rate was 29.4% in 192,814 patients with aspiration pneumonia and 11.6% in 909,078 patients with other pneumonia.^4^ In a systematic review of 19 studies, aspiration pneumonia was associated with a relative risk of 3.62 (95% CI, 2.65-4.96) for in-hospital mortality when compared with pneumonia without aspiration risk factors.^5^

Antibiotic therapy is an essential part of management for patients with aspiration pneumonia. Empiric antibiotic coverage for aspiration pneumonia has been debated and changed over time. Historically, anaerobic bacteria were thought to be the predominant pathogen in aspiration pneumonia.^1^ However, in more recent prospective studies, anaerobes were isolated in only 0.5% cases of aspiration pneumonia^6^ and 16% of isolated bacteria.^7^

Thus, the 2019 American Thoracic Society (ATS) and Infectious Diseases Society of America (IDSA) guidelines on CAP recommend to not routinely add anaerobic coverage in patients with aspiration pneumonia, and to treat with first-line antibiotics for CAP such as ceftriaxone or levofloxacin.^8^ This recommendation also considered the potential harmful consequences of broader empiric antibiotic coverage that increase risk of *Clostridioides difficile* colitis and select for antimicrobial resistance.^8^

Very little evidence is available on the comparative effectiveness of empiric antibiotic therapy with limited or extended anaerobic coverage for aspiration pneumonia. A recent systematic review found three relevant studies (two observational studies and one randomized controlled trial).^9^ All three studies did not show a significant difference in mortality or clinical cure rate with extended anaerobic coverage.^9^ However, the small sample sizes that ranged from 117 to 637 patients led to imprecise estimates with large CIs that cannot exclude clinically important differences.^9^

A large multicenter study of aspiration pneumonia that compares first-line CAP antibiotic therapy with limited vs extended anaerobic coverage is needed to conclude if any clinically important differences exist in effectiveness and adverse effects. To this end, we conducted a multicenter retrospective cohort study of patients admitted with community-acquired aspiration pneumonia to compare first-line CAP antibiotic therapy with limited vs extended anaerobic coverage in terms of in-hospital mortality and risk of *C difficile* colitis.

## Study Design and Methods

We conducted a retrospective cohort study across 18 hospitals in Ontario, Canada, and adopted a target trial methodologic approach.^10^ The Unity Health Toronto Research Ethics Board approved this study (Identifier: SMH REB 20-216). The study was reported according to the Strengthening the Reporting of Observational Studies in Epidemiology guidelines.^11^

### Data Source

This study used the GEMINI database of internal medicine inpatients that included administrative and clinical data linked at the patient level.^12^^,^^13^ Administrative data included demographics, diagnoses, interventions, discharge destination, and readmission during initial emergency room visit and hospital stay.^12^ The International Statistical Classification of Diseases and Related Health Problems, 10th Revision, Canada (ICD-10-CA) was used to classify diagnoses before, during, and after hospital admission.^14^ Medication orders and blood work results were collected from hospital electronic information systems.^12^

### Eligibility Criteria

Consecutive adult patients admitted to the medical inpatient service for aspiration pneumonia at 18 acute care hospitals in Ontario, Canada, from January 1, 2015, to January 1, 2022, were included in this study. To be eligible, the physician needed to have made a diagnosis of aspiration pneumonia and to have treated the patient with antibiotics used for CAP. The sample size was based on the chosen study date range, which was limited by the data available in the GEMINI database.

Physician diagnosis of aspiration pneumonia was based on the most responsible discharge diagnosis of aspiration pneumonia as reported by hospitals to the Canadian Institute of Health Information Discharge Abstract Database using the ICD-10-CA diagnosis code J69.0. Code J69.0 refers to pneumonitis resulting from food and vomit including aspiration pneumonia not otherwise specified or resulting from food, gastric secretions, milk, or vomit.^14^

A physician’s intention to treat aspiration pneumonia with antibiotic therapy was based on initiation of a first-line antibiotic parenterally within 2 days of admission according to the 2019 ATS and IDSA guidelines.^8^ The first-line antibiotic for CAP could be ceftriaxone, cefotaxime, amoxicillin-clavulanate (equivalent to ampicillin-sulbactam, which was not available in Canada), levofloxacin, or moxifloxacin.^8^

Patients were excluded if they fulfilled any of following criteria:

1. The patient died before receiving antibiotic treatment because they would not have been included in the analogous trial.
2. The patient had a diagnosis of lung abscess or empyema based on ICD-10-CA diagnosis codes J85.x and J86.x, which would require extended anaerobic coverage.^8^
3. The patient received only oral antibiotics, because this would introduce confounding by indication. Patients given only oral antibiotics would be more likely to receive extended anaerobic coverage, because all first-line CAP antibiotics with extended anaerobic coverage (amoxicillin-clavulanate and moxifloxacin) can be given orally, whereas some antibiotics with limited anaerobic coverage (ceftriaxone and cefotaxime) can be given only parenterally. Oral route of administration also was associated with outcome because less severely ill patients would be more likely to be treated with oral antibiotics initially. In clinical practice, patients with aspiration pneumonia usually are initiated on parenteral antibiotics because they typically have swallowing difficulties or are kept nil per os during the acute event.

This study included only community-acquired aspiration pneumonia based on diagnosis and treatment within the first 2 days of admission. Hospital-acquired aspiration pneumonia was not included in this study because it is a different clinical entity in terms of microbiology, empiric antibiotic therapy, and prognosis.

### Treatment Strategies

Patients were classified into a limited anaerobic coverage (LAC) group and extended anaerobic coverage (EAC) group based on the initial antibiotic they received within 2 days of admission. Ceftriaxone, cefotaxime, and levofloxacin were defined as having LAC because they cover some oral anaerobes such as *Peptostreptococcus* species.^15^ Amoxicillin-clavulanate, moxifloxacin, metronidazole, and clindamycin were defined as having EAC because they cover most oral and gut anaerobes including the *Bacteroides fragilis* group.^15^ Thus, patients in the LAC group received ceftriaxone, cefotaxime, or levofloxacin monotherapy. Patients in the EAC group received amoxicillin-clavulanate, moxifloxacin, or any of ceftriaxone, cefotaxime, or levofloxacin in combination with an EAC antibiotic such as clindamycin or metronidazole. Using a target trial approach, the index time was the time when the patient received the first dose of antibiotic regimen, which would be analogous to randomization in a trial.

### Outcomes

Patients were followed up until hospital discharge. The primary outcome was in-hospital mortality that occurred after the index time. A mortality difference of 3% was considered the minimally important difference based on the noninferiority margin of 3% for mortality in published CAP antibiotic trials.^16^^,^^17^ Secondary outcomes included incident diagnoses of *C difficile* colitis after admission, time to being discharged alive, and readmission to the medical or intensive care services of participating hospitals within 30 days of discharge. Exploratory outcome included 30-day attributable mortality, which was defined as death within 30 days after initially being admitted for aspiration, pneumonia, or both. This also would include patients who were discharged and then readmitted to a GEMINI hospital site with aspiration, pneumonia, or both and subsequently died. This would not have captured deaths outside the hospital.

### Assignment Procedures

Covariates were prognostic factors before index time, which included the following: demographics, including age, sex, and residence in a long-term care home; hospital admission, including hospital site, admission year, and admission meteorological season; comorbidities, including updated Charlson Comorbidity Index^18^; and illness severity, including ICU admission within 48 h of admission, modified Laboratory-Based Acute Physiology Score (mLAPS) within 24 h of admission based on laboratory parameters (sodium, BUN, creatinine, albumin, hematocrit, WBC count, arterial pH, arterial Paco_2_, arterial Pao_2_, glucose, and bilirubin).^19^ Higher mLAPS signified higher illness severity.^19^ In a prior study, mLAPS performed as well as CURB-65 score in predicting mortality for CAP.^20^

### Statistical Analysis

Complete case analysis was performed. For descriptive analysis of continuous variables, mean ± SD or median (interquartile range [IQR]) were used when appropriate. Counts and percentages were used to describe categorical variables. Absolute standardized difference of the mean was used to describe the balance of baseline characteristics. A meaningful difference was defined as a standardized difference of > 0.1.^21^

In keeping with a target trial approach, a modified intention-to-treat analysis was performed. To be included in this analysis, patients must have been receiving the same antibiotic for > 1 day or until death or discharge. Because most antibiotics were dosed daily, this ensured that patients received more than a single antibiotic dose. If patients were switched to another antibiotic regimen after 2 days, then they were analyzed based on the initial antibiotic group. A per-protocol analysis also was performed that included only patients who completed the antibiotic regimen they were prescribed initially without switching to or adding on another antibiotic during hospital stay.

For outcomes other than time to discharge, a risk difference with CI^22^ was calculated as the risk for EAC group minus risk for LAC group. For time to discharge, a competing risk model was used. Possible end points in this model included being discharged alive or dying in hospital. The Fine and Gray^23^ model was used to estimate the subdistribution hazard ratio (sHR) for being discharged alive.

Measured covariates before the index time were balanced between the two groups using a propensity score-based method. Propensity scores were estimated using a logistic regression of the covariates and then were balanced using overlap weighting.^24^ Overlap weighting calculates the average treatment effect for the overlap population, which is the population with similar covariates distribution for which clinical equipoise exists and patients would be eligible for the analogous trial.^24^^,^^25^ Overlap weighting for two groups will always lead to an exact balance in the means of any included covariates, leading to an absolute standardized difference of 0.^24^^,^^25^ The weighted difference in means for outcomes would be the average risk difference. The overlap weights were entered into the competing risk model to estimate an adjusted sHR for being discharged alive.

A sensitivity analysis was carried out that accounted for hospital sites as clusters using a generalized linear mixed-effect model.^26^ Reported CIs were all two-sided 95% CIs. R version 4.1.3 software (R Foundation for Statistical Computing) was the statistical software used, and the PSweight package was used for overlap weighting of propensity scores.^27^

### Data Reporting

To protect patient confidentiality, all cells containing or revealing five individuals or fewer were suppressed according to GEMINI data policy.

## Results

**Figure 1:**
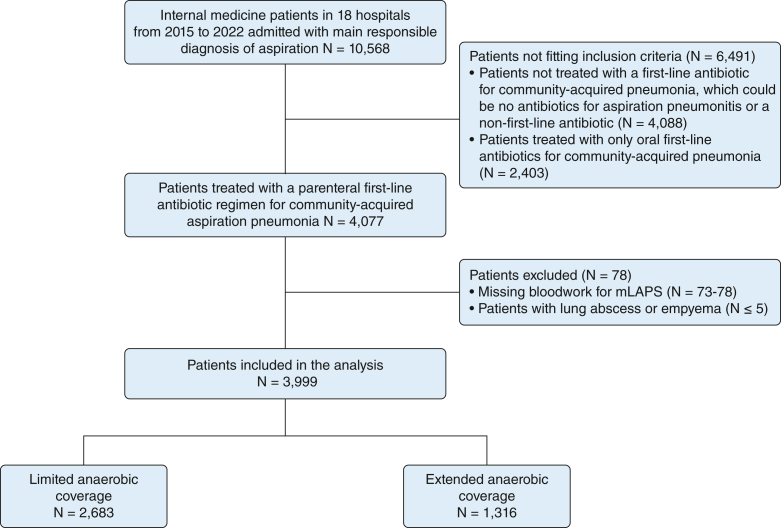
Flow diagram showing patient disposition. Numbers for each exclusion criteria given as a range because of suppression of cells with five or fewer patients. mLAPS = modified Laboratory-Based Acute Physiology Score.

Of 3,999 patients included in the study, 2,683 patients (67.1%) and 1,316 patients (32.9%) were in the LAC and EAC groups, respectively (Fig 1). The antibiotics used in each group are described in e-Table 1. The median duration of receiving antibiotics was 5 days (IQR, 3-7 days) and 7 days (IQR, 4-8 days) in the LAC and EAC groups, respectively. The excluded patients treated with only oral antibiotics are described in e-Table 2. These patients were younger and showed less severe illness based on ICU admission as well as mLAPS, leading to a much lower in-hospital mortality.

### Baseline Characteristics

Baseline characteristics are described in Table 1. Of note, the proportion of patients in each group differed significantly across hospital sites and admission year. The proportion of patients with LAC steadily increased from 2015 to 2021, which likely reflected the increasing adoption of the ATS and IDSA guidelines.^8^ Other patient baseline characteristics were well balanced even before propensity score adjustment. The organisms causing aspiration pneumonia based on ICD-10-CA codes were specified in only 17 patients (0.4%). Organisms included *Klebsiella* species, *Escherichia coli*, other gram-negative bacilli, and *Staphylococcus aureus*, which were all identified in fewer than five patients.

**Table 1:** Baseline Characteristics

Characteristic	Limited Anaerobic Coverage (n = 2,683)	Extended Anaerobic Coverage (n = 1,316)	ASDM
Age, y	79.6 ± 14.7	79.7 ± 14.7)	0.0016
Sex			
Female	1,100 (41.0)	514 (39.1)	0.0396
Male	1,583 (59.0)	802 (60.9)	0.0396
From long-term care home	637 (23.7)	274 (20.8)	0.0702
Hospital site			
A	181 (6.8)	49 (3.7)	0.1360
B	238 (8.9)	76 (5.8)	0.1190
C	≤ 5 (≤ 0.2)	≤ 5 (≤ 0.4)	0.0473
D	133 (5.0)	23 (1.8)	0.1790
E	86 (3.2)	19 (1.4)	0.1171
F	185 (6.9)	110 (8.4)	0.0552
G	239 (8.9)	116 (8.8)	0.0033
H	99 (3.7)	10 (0.8)	0.1996
I	147 (5.5)	14 (1.1)	0.2501
J	165 (6.2)	133 (10.1)	0.1452
K	356 (13.3)	130 (9.9)	0.1061
L	292 (10.9)	305 (23.2)	0.3315
M	19 (0.7)	≤ 5 (≤ 0.4)	0.0570
N	58 (2.2)	6 (0.5)	0.1505
O	183 (6.8)	39 (3.0)	0.1795
P	136 (5.1)	36 (2.7)	0.1207
Q	≤ 5 (≤ 0.2)	≤ 5 (≤ 0.4)	0.0546
R	159 (5.9)	246 (18.7)	0.3961
Admission year			
2015	228 (8.5)	212 (16.1)	0.2333
2016	296 (11.0)	333 (25.3)	0.3766
2017	403 (15.0)	239 (18.2)	0.0845
2018	437 (16.3)	227 (17.3)	0.0257
2019	484 (18.0)	137 (10.4)	0.2197
2020	483 (18.0)	114 (8.7)	0.2774
2021	352 (13.1)	54 (4.1)	0.3256
Admission season			
Spring	689 (25.7)	372 (28.3)	0.0583
Summer	655 (24.4)	294 (22.3)	0.0490
Autumn	666 (24.8)	320 (24.3)	0.0118
Winter	673 (25.1)	330 (25.1)	0.0002
Charlson Comorbidity Index	1.2 ± 1.5	1.2 ± 1.5	0.0260
Heart failure	231 (8.6)	124 (9.4)	0.0284
COPD	108 (4.0)	61 (4.6)	0.0300
Neoplasm	43 (1.6)	28 (2.1)	0.0388
Liver disease	13 (0.5)	7 (0.5)	0.0067
Dementia	395 (14.7)	213 (16.2)	0.0405
Prior stroke	21 (0.8)	13 (1.0)	0.0219
Chronic kidney disease	15 (0.6)	14 (1.1)	0.0563
Illness severity			
ICU admission	323 (12.0)	157 (11.9)	0.0033
mLAPS on admission	24.0 ± 15.7	25.3 ± 15.8	0.0768
CURB65 score^a^	2.2 ± 1.0 (n = 261)	2.2 ± 0.8 (n = 76)	0.0155

Data are presented as No. (%) or mean ± SD unless otherwise indicated. ASDM = absolute standardized difference of the mean; mLAPS = modified Laboratory-Based Acute Physiology Score.

a CURB-65 score was not available for most patients because acute confusion was not captured reliably in the GEMINI database for most hospitals and urea was not routinely analyzed in some hospitals.

### Outcomes

**Figure 2:**
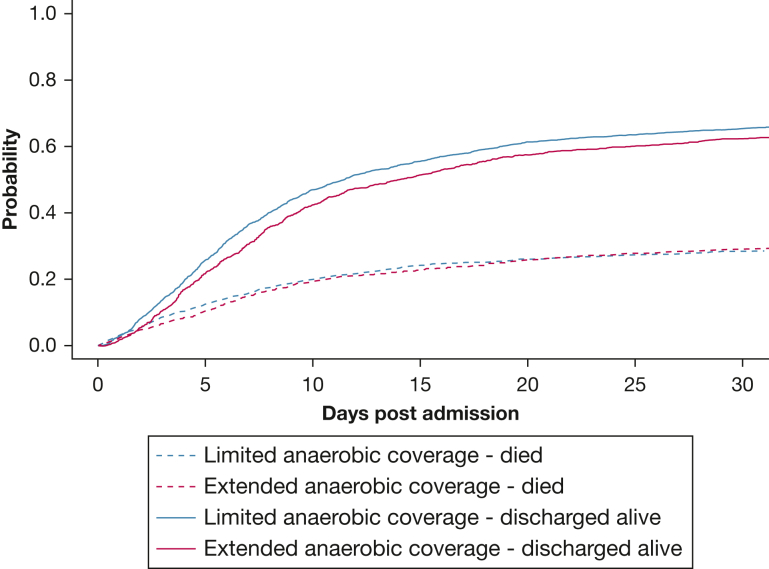
Graph showing cumulative incidence curves.

The median length of stay was 6.7 days (IQR, 3.4-12.7 days) and 7.6 days (IQR, 4.0-15.4 days) in the LAC and EAC groups, respectively. Eight hundred fourteen patients (30.3%) and 422 patients (32.1%) died in the hospital in the LAC and EAC groups, respectively. The in-hospital mortality based on antibiotic classes and CURB-65 score are shown in e-Table 3 and e-Figure 1, respectively. The cumulative incidence curves for being discharged alive and dying in hospital are shown in Figure 2. In a competing risk model, EAC showed an sHR of 0.92 (95% CI, 0.85-0.99) for being discharged alive. Kaplan-Meier survival curve for 30-day attributable mortality is shown in e-Figure 2. The outcomes are described in Table 2. Of those discharged alive, 345 of 1,869 patients (18.5%) and 164 of 894 patients (18.3%) in the LAC and EAC groups, respectively, were readmitted within 30 days.

**Table 2:** Primary and Secondary Outcomes

Variable	Limited Anaerobic Coverage (n = 2,683)	Extended Anaerobic Coverage (n = 1,316)	Extended Anaerobic Coverage vs Limited Anaerobic Coverage
Primary outcome			
In-hospital mortality	814 (30.3%)	422 (32.1%)	RD, 1.7% (95% CI, –1.3% to 4.8%) aRD, 1.6% (95% CI, –1.7% to 4.9%)
Secondary outcomes			
Transfer to ICU	66 (2.5%)	35 (2.7%)	RD, 0.2% (95% CI, –0.8% to 1.3%) aRD, 0.5% (95% CI, –0.6% to 1.7%)
*C difficile* colitis	≤ 5 (≤ 0.2%)	11-15 (0.8%-1.1%)	RD, 0.8% (95% CI, 0.3%-1.4%) aRD, 1.0% (95% CI, 0.3%-1.7%)
Exploratory outcome			
30-d attributable mortality^a^	781 (29.1%)	394 (29.9%)	RD, 0.8% (95% CI, –2.2% to 3.9%) aRD, 0.9% (95% CI, –2.3% to 4.2%)

Data are presented as No. (%) unless otherwise indicated. aRD = adjusted risk difference after overlap weighting of propensity score; RD = risk difference.

a Lower than the in-hospital mortality because it excluded patients who died after a hospital stay of > 30 d.

### Adjustment Using Propensity Score

Table 3 describes the population after overlap weighting using propensity scores. The outcomes within this overlap population are described in Table 2. For in-hospital mortality, the adjusted risk difference for EAC minus LAC was 1.6% (95% CI, –1.7% to 4.9%). In a competing risk model, the EAC group showed an adjusted sHR of 0.92 (95% CI, 0.84-1.00) for being discharge alive. For *C difficile* colitis, the adjusted risk difference was 1.0% (95% CI, 0.3%-1.7%).

**Table 3:** Baseline Characteristics After Overlap Weighting Using Propensity Scores

Characteristic	Limited Anaerobic Coverage (Effective Sample Size = 1,837.4)	Extended Anaerobic Coverage (Effective Sample Size = 1,130.4)
Age, y	79.6 ± 14.4	79.6 ± 15.1
Sex		
Female	39.8	39.8
Male	60.2	60.2
From long-term care home	22.2	22.2
Hospital site		
A	5.3	5.3
B	7.6	7.6
C	0	0
D	2.6	2.6
E	2.0	2.0
F	9.5	9.5
G	10.1	10.1
H	1.3	1.3
I	1.8	1.8
J	8.9	8.9
K	12.0	12.0
L	17.4	17.4
M	0.5	0.5
N	0.8	0.8
O	4.3	4.3
P	4.0	4.0
Q	0	0
R	11.8	11.8
Admission year		
2015	13.1	13.1
2016	18.7	18.7
2017	18.0	18.0
2018	18.3	18.3
2019	13.7	13.7
2020	11.9	11.9
2021	6.3	6.3
Admission season		
Spring	27.0	27.0
Summer	22.8	22.8
Autumn	25.0	25.0
Winter	25.2	25.2
Charlson comorbidity index	1.2 ± 1.5	1.2 ± 1.5
Illness severity		
ICU admission	11.9	11.9
mLAPS on admission	24.8 ± 15.6	24.8 ± 16.0

Data are presented as percentage or mean ± SD. mLAPS = modified Laboratory-Based Acute Physiology Score.

### Additional Analyses

Results from sensitivity analysis that considered hospital sites as clusters are described in e-Table 4, and the per-protocol analysis results are described in e-Table 5, which are all similar to the main analysis results as shown previously.

## Discussion

In this retrospective cohort study of patients admitted with community-acquired aspiration pneumonia across 18 hospital sites, in-hospital mortality was not significantly different between EAC and LAC groups (adjusted risk difference, 1.6%; 95% CI, –1.7% to 4.9%). The lower CI limit of –1.7% excluded a clinically important difference of 3% mortality reduction.^16^^,^^17^ Thus, extended anaerobic coverage seemed unlikely to lead to a clinically important improvement in mortality. However, extended anaerobic coverage was associated with a significantly increased risk of *C difficile* colitis (adjusted risk difference, 1.0%; 95% CI 0.3%-1.7%). These findings suggest that extended anaerobic antibiotic coverage did not offer any additional benefit for aspiration pneumonia and could have increased the risk of harm.

Our study findings are consistent with those from a small randomized controlled trial^28^ and three prior observational studies.^29^, ^30^, ^31^ Similar to these four studies, our study did not show a significant difference between LAC and EAC groups in terms of mortality. Unlike the prior three studies,^28^^,^^30^^,^^31^ our study included only patients receiving first-line antibiotic agents for CAP. In addition, our study showed a much larger sample size that was more than four times the sample size of all four studies combined. This allowed for a more precise estimate with a narrow 95% CI to exclude a clinically important difference.

Our study findings have important implications. This study adds strong real-world clinical evidence to the ATS and IDSA 2019 CAP guidelines recommendation of not routinely adding anaerobic coverage for aspiration pneumonia.^8^ Patients with community-acquired aspiration pneumonia can be treated with ceftriaxone or levofloxacin without adding another antibiotic for anaerobic coverage. The avoidance of unnecessary antibiotic administration can decrease the risk of antibiotic adverse effects, especially *C difficile* colitis. On a larger scale, limiting unnecessary antibiotic use may lower antibiotic selective pressure and may result in less antibiotic resistance. In hospitals, antimicrobial stewardship programs can implement targeted interventions to de-escalate antibiotic therapy for aspiration pneumonia.

Our study has several strengths. First, the large sample size of 3,999 patients allowed for more precise estimates. Second, the study included 18 hospitals that included both academic and community hospitals, thereby increasing the generalizability of the study findings. Third, the GEMINI database was comprehensive and included detailed medication and patient data. Data on medication orders allowed accurate capture of antibiotic exposure during the entire hospital stay. Patient data allowed for adjustment of many prognostic factors in the propensity score overlap weighting analysis. All study patients underwent complete follow-up in hospital.

The study had important limitations that merit mentioning. First, ICD-10-CA codes for aspiration pneumonia have not been validated previously to capture aspiration pneumonia and may have included aspiration pneumonitis that does not require antibiotic therapy.^32^ However, we coupled the ICD-10-CA diagnosis codes with the physician’s decision to treat with first-line CAP antibiotics, which reflected clinical diagnosis of aspiration pneumonia necessitating antibiotic therapy. Most aspiration pneumonitis cases resolve within 48 h.^32^^,^^33^ Based on the cumulative incidence curves (Fig 2), mortality continued to increase steadily after day 2, which followed the typical course for aspiration pneumonia, rather than aspiration pneumonitis. A prior study used the same method based on International Classification of Diseases codes to capture and differentiate aspiration pneumonia from other pneumonias.^4^

Second, the primary outcome was in-hospital mortality because complete follow-up ended on discharge from hospital. We were unable to report out-of-hospital deaths or readmission to hospitals not participating in the GEMINI network. However, in-hospital mortality likely captured most of the attributable deaths resulting from aspiration pneumonia. Our in-hospital mortality rate of 30% was very close to the reported 29% 30-day all-cause mortality for aspiration pneumonia in a large nationwide cohort study of > 1 million patients,^4^ suggesting that our study did not miss a significant number of deaths. Patients who were discharged, deteriorated, and then readmitted to a GEMINI hospital site still were captured in our study using the exploratory outcome of 30-day attributable mortality, which was similar between the two groups. This should capture the vast majority of readmissions, because a prior study showed that > 80% of readmissions occur at the same hospital^34^ and our database accounted for readmission to any hospital within the GEMINI network that make up roughly one-half of all acute-care hospital beds in Ontario.

Third, we were able to capture new *C difficile* colitis diagnosed only in hospital, so diagnoses made after discharge were not captured. The risk of *C difficile* colitis is estimated to be highest within 3 to 14 days from the first antibiotic dose,^35^ and then decreases over time.^36^ Thus, our study would have captured the highest risk period during a patient’s hospital stay.

Fourth, as is the case for any observational study, residual confounding may still be present. It is plausible that clinicians’ empiric antibiotic choice depended on illness severity, where sicker patients were more likely to receive additional anaerobic coverage. This did not seem to be the case in this study based on baseline characteristics between the two groups with similar proportion of ICU admissions and mLAPS (Table 1). We had also balanced many prognostic factors using propensity score overlap weighting.

Fifth, a bacterial pathogen was not identified in the vast majority of study patients. ICD-10-CA codes likely are highly insensitive for specific bacterial pathogens. As well, the microbiological yield in aspiration pneumonia is low, because it is reasonable to treat moderately severe aspiration pneumonia without microbiological workup based on the current guidelines.^8^ Furthermore, even with a full microbiological workup, anaerobes are difficult to grow in cultures.^6^ Our study reflects the real-world clinical practice in which almost all cases of aspiration pneumonia are treated with antibiotics empirically without available microbiological data to tailor antibiotic therapy.

## Interpretation

In conclusion, our study adds to the existing evidence that extended anaerobic coverage likely is unnecessary in community-acquired aspiration pneumonia and is associated with a greater risk of *C difficile* colitis. It is reasonable to treat these patients with a first-line antibiotic therapy for CAP such as ceftriaxone without adding clindamycin or metronidazole.

## Funding/Support

The authors have reported to *CHEST* that no funding was received for this study. The development of the GEMINI data platform was supported with funding from the Canadian Cancer Society, the Canadian Frailty Network, the Canadian Institutes of Health Research, the Canadian Medical Protective Association, Green Shield Canada Foundation, the Natural Sciences and Engineering Research Council of Canada, Ontario Health, the 10.13039/100014608St. Michael’s Hospital Foundation, the St. Michael’s Hospital Association Innovation Fund, the University of Toronto Department of Medicine, and in-kind support from partner hospitals and the Vector Institute. A. A. V. receives salary support as the Temerty Professor of Artificial Intelligence Research and Education at the University of Toronto.

## Financial/Nonfinancial Disclosures

None declared.

## Supplementary Data

e-Online Data
