# Supplementary material for: Anaerobic Antibiotic Coverage in Aspiration Pneumonia and the Associated Benefits and Harms: A Retrospective Cohort Study
Source: Chest. 2024 Feb 20;166(1):39–48. doi: 10.1016/j.chest.2024.02.025 (PMC11251078; doi:10.1016/j.chest.2024.02.025)
Supplement: e-Online Data [file mmc1.docx]

e-Table 1. Antibiotics used within each group

|  | Limited anaerobic coverage  (N=2,683) | Extended anaerobic coverage  (N=1,316) |
| --- | --- | --- |
| Beta-lactams |  |  |
| Amoxicillin-Clavulanate | ≤5 (≤0.2%) | 6 (0.5%) |
| Cefotaxime | ≤5 (≤0.2%) | ≤5 (≤0.4%) |
| Ceftriaxone | 2,612 (97.4%) | 660 (50.2%) |
| Fluoroquinolones |  |  |
| Levofloxacin | 70 (2.6%) | 9 (0.7%) |
| Moxifloxacin | 0 (0%) | 640 (48.6%) |
| Antibiotics for anaerobic coverage |  |  |
| Clindamycin | 0 (0%) | 32 (2.4%) |
| Metronidazole | 0 (0%) | 664 (50.5%) |
| Antibiotics for atypical coverage |  |  |
| Macrolides | 767 (28.6%) | 160 (12.2%) |

e-Table 2. Excluded patients with aspiration pneumonia treated with oral antibiotics initially

|  | Oral antibiotics  (N=2,403) | Parenteral antibiotics  (N=3,999) |
| --- | --- | --- |
| Extended anaerobe coverage | 2,348 (97.7%) | 1,316 (32.9%) |
| Age in years  Mean (SD) | 77.0 (16.3) | 79.7 (14.7) |
| Sex |  |  |
| Female | 931 (38.7%) | 1,614 (40.4%) |
| Male | 1,472 (61.3%) | 2,385 (59.6%) |
| From long-term care home | 464 (19.3%) | 911 (22.8%) |
| Charlson comorbidity Index  Mean (SD) | 1.1 (1.5) | 1.2 (1.5) |
| Illness severity |  |  |
| ICU admission | 192 (8.0%) | 480 (12.0%) |
| mLAPS on admission  Mean (SD) | 21.6 (14.3) | 24.4 (15.8) |
| Primary outcome |  |  |
| In-hospital mortality | 242 (10.1%) | 1,236 (30.9%) |

e-Table 3. In-hospital mortality rate for antibiotic classes

|  | In-hospital mortality rate |
| --- | --- |
| Limited anaerobic coverage group |  |
| Ceftriaxone or Cefotaxime alone | 795 (30.4%) / 2,613 |
| Levofloxacin alone | 19 (27.1%) / 70 |
| Extended anaerobic coverage group |  |
| Amoxicillin-Clavulanate | ≤5 (≤83.3%) / 6 |
| [Ceftriaxone or Cefotaxime] plus Metronidazole | 201 (33.1%) / 608 |
| [Ceftriaxone or Cefotaxime] plus an antibiotic with extended anaerobe coverage that is not Metronidazole | 9 (17.0%) / 53 |
| Levofloxacin plus an antibiotic with extended anaerobe coverage | ≤5 (≤55.6%) / 9 |
| Moxifloxacin | 209 (32.7%) / 640 |

e-Table 4. Primary and secondary outcomes after adjustment by overlap weighting of propensity scores with hospital sites as clusters

|  | Comparison  Extended anaerobic coverage vs.  Limited anaerobic coverage |
| --- | --- |
| Primary outcome |  |
| In-hospital mortality | aRD: 1.6% (95% CI -1.8% to 5.0%) |
| Secondary outcomes |  |
| Transfer to ICU | aRD: 0.5% (95% CI -0.7% to 1.7%) |
| *Clostridioides difficile* colitis | aRD: 1.0% (95% CI 0.3% to 1.7%) |
| Exploratory outcome |  |
| 30-day attributable mortality | aRD: 0.9% (-2.4% to 4.3%) |

aRD = adjusted risk difference after overlap weighting of propensity score

e-Table 5. Per-protocol analysis

|  | Limited anaerobic coverage  (N=2,337) | Extended anaerobic coverage  (N=702) | Comparison  Extended anaerobic coverage vs.  Limited anaerobic coverage |
| --- | --- | --- | --- |
| Primary outcome |  |  |  |
| In-hospital mortality | 718 (30.7%) | 243 (34.6%) | RD: 3.9% (95% CI 0% to 7.9%)  aRD: 5.4% (95% CI 1.0% to 9.7%) |
| Secondary outcomes |  |  |  |
| Transfer to ICU | 58 (2.5%) | 15 (2.1%) | RD: -0.3% (95% CI -1.5% to 1.1%)  aRD: -0.1% (95% CI -1.5% to 1.3%) |
| *Clostridioides difficile* colitis | ≤5 (≤0.2%) | 6 to 10  (0.9% to 1.4%) | RD: 1.0% (95% CI 0.4% to 2.1%)  aRD: 1.3% (95% CI 0.3% to 2.3%) |
| Exploratory outcome |  |  |  |
| 30-day attributable mortality | 693 (29.7%) | 237 (33.8%) | RD: 4.1% (0.2% to 8.1%)  aRD: 5.4% (1.1% to 9.7%) |

aRD = adjusted risk difference after overlap weighting of propensity score; RD = risk difference

e-Figure 1. In-hospital mortality rates by CURB65 score


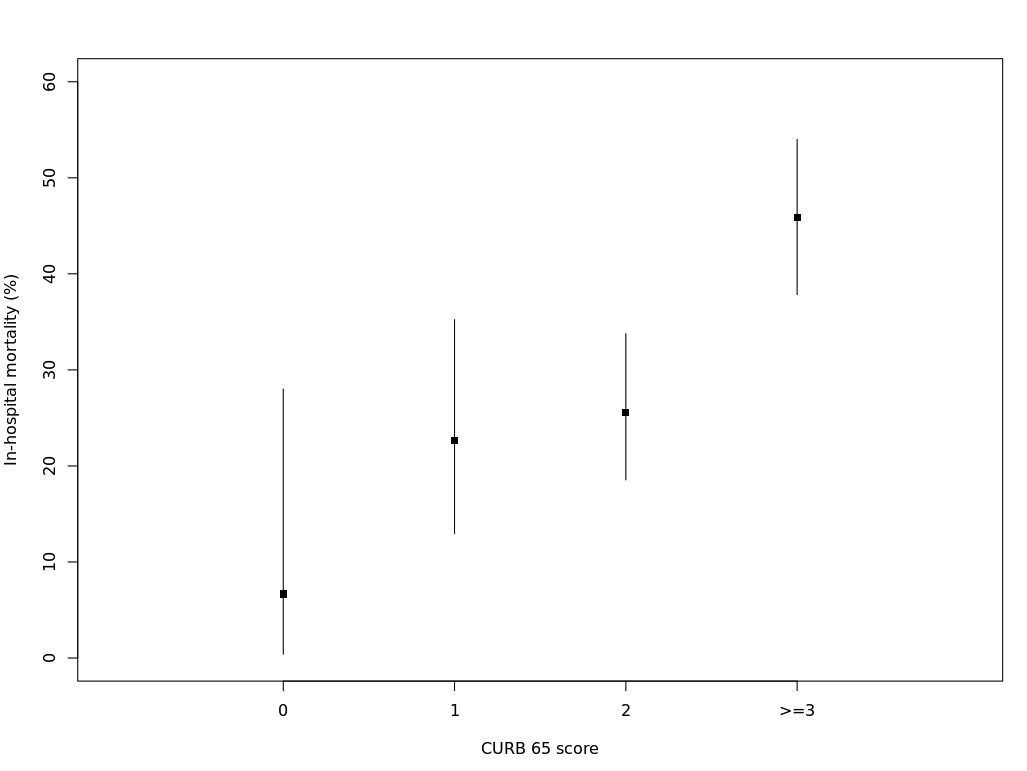


The vertical lines represent the 95% confidence interval around the point estimates

| CURB65 score | 0 point | 1 point | 2 points | >=3 points |
| --- | --- | --- | --- | --- |
| Patients who died in hospital | ≤5 (≤33.3%) | 12 (22.6%) | 32 (25.6%) | 66 (45.8%) |
| Total number of patients | 15 | 53 | 125 | 144 |

e-Figure 2. Kaplan-Meier Curve for 30-day attributable mortality


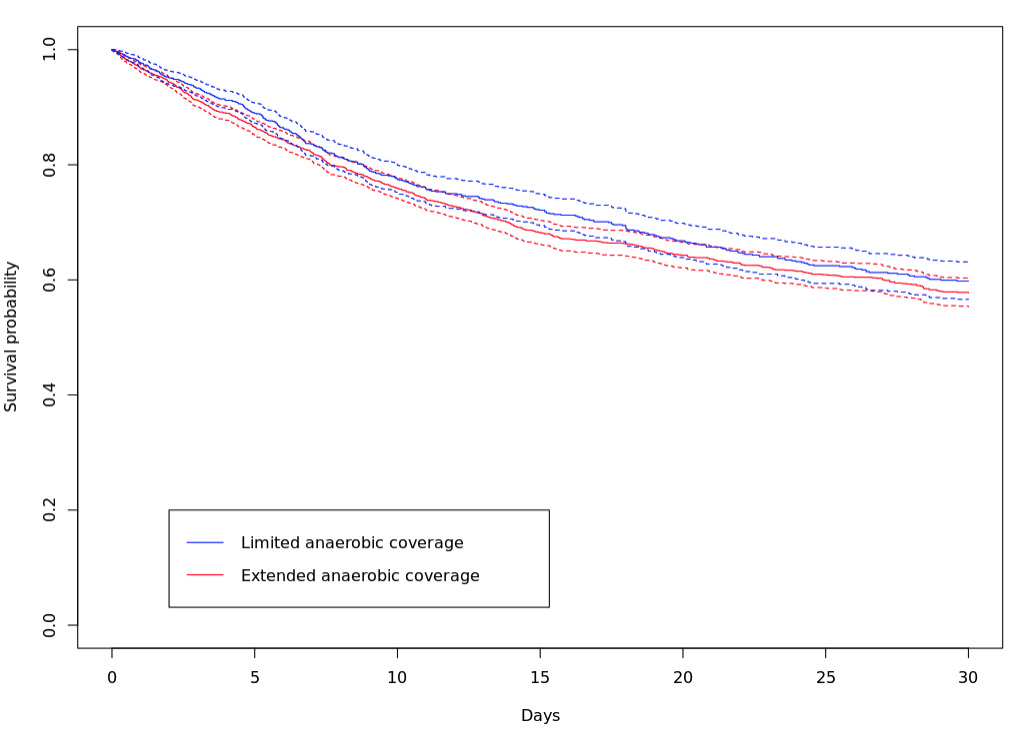


The solid line is the Kaplan-Meier survival curve and dotted lines represent the 95% confidence intervals
